# Evaluating the economic impact of water scarcity in a changing world

**DOI:** 10.1038/s41467-021-22194-0

**Published:** 2021-03-26

**Authors:** Flannery Dolan, Jonathan Lamontagne, Robert Link, Mohamad Hejazi, Patrick Reed, Jae Edmonds

**Affiliations:** 1 Department of Civil and Environmental Engineering, Tufts University, Medford, MA USA; 2 Department of Medicine, University of Virginia School of Medicine, Charlottesville, VA USA; 3 Joint Global Change Research Institute, Pacific Northwest National Laboratory, College Park, MA USA; 4 King Abdullah Petroleum Studies and Research Center, Riyadh, Saudi Arabia; 5 School of Civil and Environmental Engineering, Cornell University, Ithaca, NY USA

**Keywords:** Climate and Earth system modelling, Environmental economics, Hydrology

## Abstract

Water scarcity is dynamic and complex, emerging from the combined influences of climate change, basin-level water resources, and managed systems’ adaptive capacities. Beyond geophysical stressors and responses, it is critical to also consider how multi-sector, multi-scale economic teleconnections mitigate or exacerbate water shortages. Here, we contribute a global-to-basin-scale exploratory analysis of potential water scarcity impacts by linking a global human-Earth system model, a global hydrologic model, and a metric for the loss of economic surplus due to resource shortages. We find that, dependent on scenario assumptions, major hydrologic basins can experience strongly positive or strongly negative economic impacts due to global trade dynamics and market adaptations to regional scarcity. In many cases, market adaptation profoundly magnifies economic uncertainty relative to hydrologic uncertainty. Our analysis finds that impactful scenarios are often combinations of standard scenarios, showcasing that planners cannot presume drivers of uncertainty in complex adaptive systems.

## Abstract (web summary)

The impacts of water scarcity depend on physical basin characteristics and global economic dynamics. Here, the authors show scenario assumptions can yield either highly positive or negative economic impacts due to water scarcity, and the drivers of these impacts are basin-specific and cannot be determined a priori.

## Introduction

Global water scarcity is a leading challenge for continued human development and achievement of the Sustainable Development Goals^1,2^. While water scarcity is often understood as a local river basin problem, its drivers are often global in nature^3^. For instance, agricultural commodities (the primary source of global water consumption^4^), are often traded and consumed outside the regions they are produced^5^. These economic trade connections mean that global changes in consumption result in impacts on local water systems^6^. Likewise, local water system shocks can also propagate globally^7,8^. Water is a critical input to other sectors, such as energy, transportation, and manufacturing^9,10^, so that changes in the regional water supply or sectoral demand can propagate across sectors and scales. Continued population growth, climate change, and globalization ensure that these multi-region, multi-sector dynamics will become increasingly important to our understanding of water scarcity drivers and impacts^11^.

Quantifying water scarcity and its impacts are active and growing research areas^12^. Early and influential work in the area largely focused on supply-oriented metrics of scarcity: per-capita water availability^13^, the fraction of available water being used^14^, and more sophisticated measures that account for a region’s ability to leverage available water given its infrastructure and institutional constraints^15^. Recent work proposes indicators such as water quality^16^, green water availability^17^, and environmental flow requirements^18^ that focus on specific facets of water scarcity. Qin et al. incorporate the flexibility of current modes of consumption to identify regions where adaptation to scarcity may be relatively difficult^19^. Other recent work focuses on the water footprint of economic activity^20,21^ making it possible to identify the economic drivers of scarcity (through virtual water trade)^6,8^. Yet knowledge gaps remain concerning how the economic costs of future water scarcity will propagate between sectors and regions as society adapts to scarcity, and how the cost of this adaptation depends on uncertainties in the projections of future conditions.

From the economic perspective, water scarcity impacts arise when the difficulty of obtaining water forces a change in consumption. For instance, abundant snowmelt may be of little use to would-be farmers if barriers (cost, institutional, etc.) prevent them from utilizing it. They will be forced to go elsewhere for water or engage in other activities, and this bears an economic cost that is not reflected in conventional water scarcity metrics. When water becomes a binding constraint, societies adapt through trade and shifting patterns of production, and the cost of that adaptation is tied to the difficulty of adopting needed changes. Changing annual cropping patterns to conserve water is easier and will impact an economy less than shuttering thermal power generation during prolonged drought^19^. In a globalized economy, the impact of such adaptation cannot be assessed in a single basin or sector in isolation, as hydrologic changes in one region reverberate across sectors around the world^3,22^. Indeed, reductions in water supply in one region may increase demands for water in another, simultaneously inducing both physical scarcity and economic benefit in ways that are difficult to anticipate ex ante^23^. Our primary research question is how these dynamics will impact society in the future, and how both the magnitude and direction of those impacts depend on future deeply uncertain conditions^24^.

To address this question, we deploy a coupled global hydrologic-economic model with basin-level hydrologic and economic resolution^25^ to compute the loss (or gain) of economic surplus due to that scarcity in each basin across a range of deeply uncertain futures. Here “economic surplus” refers to the difference between the value that consumers place on a good and the producers’ cost of providing that good^26^. The surplus is a measure of the value-added, or societal welfare gained, due to some economic activity. The change in economic surplus is an appealing metric because it captures how the impact of resource scarcity propagates across sectors and regions that depend on that resource. Change in surplus has been used in past studies to assess the impacts of water policies and to understand how to efficiently allocate water in arid regions^27,28^, though it has not typically been used to analyze the impact of water scarcity itself. One exception is a study by Berritella et al., who used the loss in equivalent variation, another welfare metric, to measure the effects of restricting the use of groundwater^29^. On a broader scale, our analysis tracks the impacts of scarcity in hundreds of basins across thousands of scenarios, revealing important global drivers of local impacts that are often missed when the spatial and sectoral scope is defined too narrowly.

Global water scarcity studies depend on long-term projections of climate, population growth, technology change, and other factors that are deeply uncertain, meaning that neither the appropriate distribution nor the correct systems model is agreed upon^24,30^. Complicating matters, the coupled human-earth system is complex, exhibiting nonlinearities and emergent properties that make it difficult to anticipate important drivers in the scenario selection process. In such a case, focusing on a few scenarios, as is common in water scarcity studies, risks missing key drivers and their interactions^31^. In contrast, recent studies advocate exploratory modeling^32^ to identify important global change scenarios^33,34^. In that approach, the uncertainty space is searched broadly and coupled-systems models are used to test the implications of different assumptions on salient measures of impact across a scenario ensemble^35^. Exploratory modeling is especially important in long-term water scarcity studies, where we show that meaningful scenarios vary widely from basin-to-basin, highlighting the inadequacy of relying on a few global narrative scenarios.

By analyzing a large ensemble of global hydro-economic futures, we arrive at three key insights. First, basin-level water scarcity may be economically beneficial or detrimental depending on a basin’s future adaptive capacity and comparative advantage, but that advantage is highly path-dependent on which deeply uncertain factors emerge as the basin-specific drivers of consequential outcomes. For instance, in the Lower Colorado Basin, the worst economic outcomes arise from limited groundwater availability and high population growth, but that high population growth can also prove beneficial under some climatic scenarios. In contrast, the future economic outcomes in the Indus Basin depend largely on global land-use policies intended to disincentive land-use change in the developing world. Our second insight is that those land-use policies often incentivize unsustainable water consumption. In the case of the Indus Basin, limiting agricultural extensification results in intensification through increased irrigation that leads to unsustainable overdraft of groundwater, with similar dynamics playing out elsewhere. Third, our results show that the nonlinear nature of water demand can substantially amplify underlying climate uncertainty, so that small changes in runoff result in large swings in economic impact. This is pronounced in water-scarce basins (like the Colorado) under high-demand scenarios. Collectively, these insights suggest that understanding and accounting for the adaptive nature of global water demand is crucial for determining basin-level water scarcity’s path-dependent and deeply uncertain impacts.

## Results

### Global-to-basin impacts

**Fig. 1:**
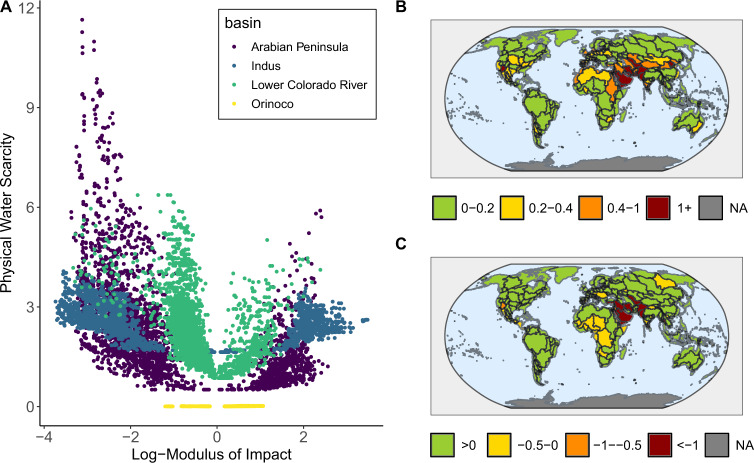
Economic and physical water scarcity. The scatter plot in panel **A** shows the two metrics in panels **B** and **C** plotted against each other in four basins. Each point represents the maximum absolute value of that metric over time in each scenario. The map in panel **B** shows WTA in each water basin while the map in panel **C** shows the log-modulus of economic impact. Both maps plot the maximum absolute value of the metric over time and the median across all scenarios. The correspondence between the two metrics is not perfect. Some water-scarce basins have more capacity to handle water scarcity and thus are not as impacted economically as others.

We calculate both physical water scarcity (Fig. 1B) and its economic impact (Fig. 1C) over the 21st century for 235 river basins for each of the 3000 global change scenarios, simulated using the Global Change Analysis Model (GCAM) integrated assessment model^36^. With the effects of inter-basin trade, hydrologic basins may experience highly positive or highly negative economic impact due to water scarcity (Fig. 1A). Here, economic impact is defined as the difference in total surplus in water markets (Supplementary Fig. 1) between a control scenario with unlimited water and an experimental scenario with limited water supply (Supplementary Fig. 2). Water scarcity usually induces negative economic impact (loss of surplus), although positive economic impact from global water scarcity can arise if a basin holds a comparative advantage over others. With this comparative advantage, a basin can become a virtual water exporter through inter-basin trade^37^, meaning it will export water-embedded goods to other regions. Though some basins experience positive impact more often than others (across the scenario ensemble), all basins experience both negative and positive impacts in some scenarios (Supplementary Table 1): no basin has a universally positive or negative outlook. As may be expected, the basins with the highest number of positive impact scenarios are those that are relatively water-rich by conventional measures (Fig. 1B), for example, the Orinoco River in northern South America (Fig. 1A).

We measure physical water scarcity using the Withdrawal-To-Availability ratio (WTA) which is computed by dividing water withdrawals by renewable supply. The correspondence between the WTA and the economic impact metric is not perfect (Fig. 1B and C). In some scenarios (for instance, those with restricted reservoir storage), basins with high physical scarcity have a small negative or even positive economic impact, and in others, basins with low physical scarcity have a negative economic impact (Fig. 1A). This highlights the importance of capturing the interdependencies between physical and economic factors that affect the welfare of a basin.

Several basins show high variance in economic impacts, including the Indus River Basin, the Arabian Peninsula, and the Lower Colorado River Basin (Supplementary Fig. 4). In addition to variance in economic impacts, those basins exhibit a wide range of physical water scarcity, are geographically diverse and are of geopolitical importance. The Orinoco Basin is also highlighted as an example of a basin that is not physically water-scarce and experiences slightly positive economic impact in most scenarios (Fig. 1A and B). Such water-rich basins are particularly well-positioned to produce more water-intensive products to offset lost production in water-scarce basins (Supplementary Fig. 3), though the stylized water markets as represented in GCAM (and indeed in all other global hydro-economic models) may overstate these benefits for some basins compared to real-world conditions. The market representation assumes that all agents have an equal opportunity to acquire water and that water is allocated in the most economically efficient manner (except for agricultural subsidies^38^). In reality, water rights frameworks and barriers to trade may block potential users from putting the water to more economically beneficial use.

The distributions of the plotted scenarios in the four selected basins (Fig. 1A) give some indication of the relationship between water scarcity and economic impact in each basin. The bi-modal spread of the scenario points (Fig. 1A) shows that higher physical water scarcity can be associated with both highly positive and severely negative economic impacts. When the distributions are wide and shallow (e.g., the Indus Basin in Fig. 1A), smaller changes in physical scarcity lead to much higher changes in economic impact compared with other basins (Table 1). This occurs if the basin cannot easily supplement renewable supply with other water sources and the price of water rises precipitously. Shifts in demand subject to these high prices lead to large swings in economic impact.

**Table 1:** Quantiles of surplus change and physical water scarcity in the four selected basins (billions US 2020$).

Hydrologic basin	10% surplus change	90% surplus change	10% WTA	90% WTA
Indus	−2010	405	0.005	2.13
Arabian Peninsula	−530	101	0.32	2.5
Lower Colorado River	−16.4	3.08	0.15	1.86
Orinoco	−2.84	4.96	0.001	0.007

The direction of shifts in demand depends on a basin’s comparative advantage (or disadvantage) due to the scenario assumptions and how these assumptions affect other basins around the world. As evidenced by the positive scenarios in water-scarce basins in Fig. 1, this comparative advantage can arise from mechanisms other than abundant water supply (e.g., higher agricultural productivity, different dietary or technological preferences, or a lower population). The equilibrium demand over the renewable supply (the WTA) could be the same in two scenarios with very different economic impacts depending on if the scenario assumptions enable a basin’s comparative advantage in one but are detrimental in another (Fig. 1A). Influential factors that determine economic impact are basin-specific (examples given in the next section). The changes in demand and resulting impacts due to these factors underscore the importance of projecting basin-level scarcity in a global context that allows for market adaptation.

### Climate system uncertainty amplification

**Fig. 2:**
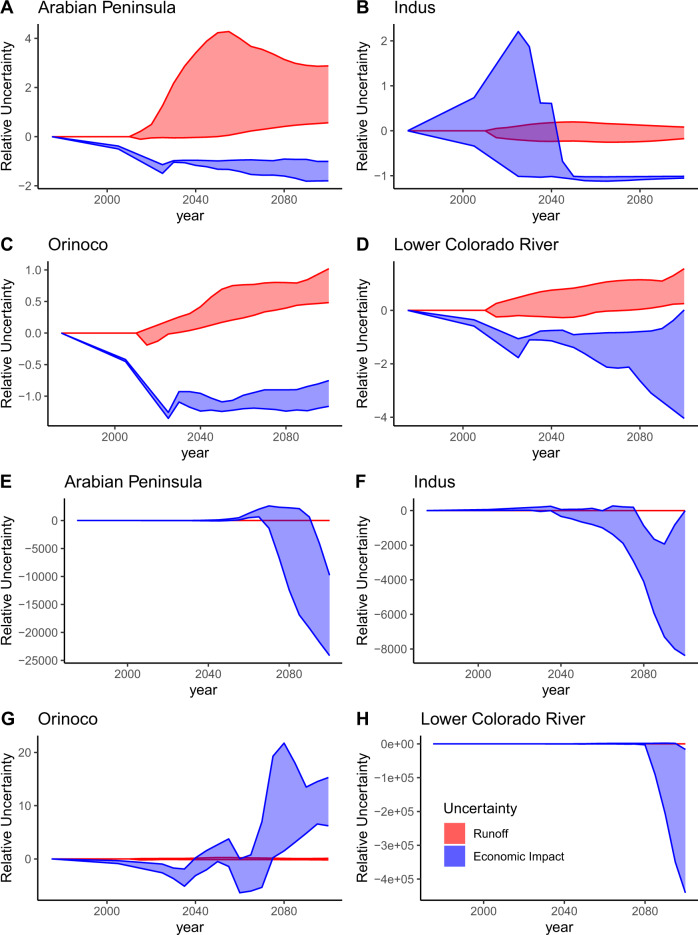
Runoff and economic impact uncertainty. Uncertainty over time plots of the four chosen basins. Values are taken relative to the 2015 baseline. Uncertainty prior to 2015 is illustrative only. The scenario group shown in **A**–**D** has the lowest mean climate-induced economic impact uncertainty over time out of the 600 groups. The scenario group shown in **E**–**H** has the highest mean climate-induced economic impact uncertainty over time. In most scenarios, runoff uncertainty is amplified by the human system, leading to higher uncertainty in economic impact.

The market response to water scarcity within a hydrologic basin usually amplifies the uncertainty in hydro-climatic projections (Fig. 2, Supplementary Fig. 5), leading to higher changes in economic impact. Analysis of the scenario ensemble revealed that differences in Earth System Model (ESM) forcing often determines the sign of impact (SA Figs. 6–9). The ESMs contribute precipitation and temperature projections to the hydrologic model used by GCAM, generating water runoff estimates (see “Methods” section). Surface water supply fluctuations heavily affect changes in economic surplus within these hydrologic basins. Other important factors include reservoir expansion (in Arabia and the Orinoco), land-use scenario (in the Indus and Orinoco), and agricultural productivity (in Arabia, the Indus, and the Orinoco) (Supplementary Figs. 6–9).

Climate uncertainty is one dimension over which decision-makers have very little control (as opposed to socioeconomic trajectories, agricultural advancements, reservoir storage, etc.). To isolate the uncertainty in the economic impact due to this fundamental climate uncertainty, 600 groups of five scenarios were created by holding all factors constant, except the ESM (of which 5 were considered). The difference between the maximum impact in this group of five and the minimum is one measure of climate-induced impact uncertainty. This uncertainty is plotted in blue in Fig. 2 compared to the runoff uncertainty in red. We find that the economic impact uncertainty is usually higher than the runoff uncertainty (Supplementary Fig. 5). Here, runoff uncertainty is the difference between the maximum runoff and the minimum runoff in the set of five scenarios. Peaks and troughs in Fig. 2 correspond to slight deviations in climate forcing in the ESMs. This in turn leads to differences in the runoff, which changes the unit costs of water, causing market adaptations and thus amplifying the economic surplus change (Supplementary Fig. 10).

High economic impact uncertainty relative to runoff uncertainty indicates that the market is very sensitive to changes in water supply. In high-demand scenarios (e.g., those with a high population and high food demand), the price of water steeply rises when shifting toward nontraditional water sources such as non-renewable reserves and desalination (Supplementary Fig. 11A). When this occurs, deviations in supply lead to highly nonlinear impacts (Fig. 2E–H). Vulnerable basins in these high-demand scenarios see steep and rapid declines in economic impact (Fig. 2E, H). Scenarios where the economic impact continues dropping through the end of the century are of particular concern and suggest that a basin no longer has the economic capacity to stabilize these negative impacts. We will henceforth call this loss in capacity an ‘economic tipping point’.

Importantly, the conditions that lead to tipping points can vary substantially across basins. For instance, in the Arabian Peninsula, tipping point conditions include low groundwater availability and pricing carbon emissions from all sectors (see “Methods” section). Even with ample groundwater supply, tipping points can occur with high population and low GDP (SSP 3 socioeconomic assumptions) in addition to pricing carbon emissions from all sectors. In some scenarios, we can see that the Arabian Peninsula experiences a positive impact mid-century by relying on relatively inexpensive water resources. After these resources run out subject to the constraints, the economic impact becomes more negative until the end of the century (Fig. 2A) and the basin utilizes an increasing amount of desalinated water (Supplementary Fig. 11B). The lack of perfect foresight within GCAM helps explain this short-term thinking, though historically the area has withdrawn groundwater at unsustainable rates^39^.

Meanwhile, the Lower Colorado River Basin experiences an economic tipping point when there is low groundwater availability, low agricultural productivity (SSP 3 agriculture and land use assumptions), and high wealth socioeconomic trajectories (SSP 5 socioeconomics). The uncertainty in economic impact in the Lower Colorado Basin is the highest out of all of the highlighted basins (Fig. 2C) and is one of the basins with the highest uncertainty in economic impact in the world.

Importantly, the factors that cause economic tipping points in these basins are not the same, nor do they always follow a well-defined global narrative such as the canonical SSPs. Table 2 shows the basins with the most highly negative impact values out of all the time periods in every scenario. Most of these scenarios contain a mixture of SSP elements (e.g., SSP 5 socioeconomics and SSP 4 agriculture in the Sabarmati). There are noticeable trends in the factors, for instance, high wealth socioeconomic trajectories (SSP 5) and the Universal Carbon Tax often lead to tipping points. However, the factors are not all the same in each basin (e.g., in the Ganges-Brahmaputra).

**Table 2:** The top five basins with the most highly negative economic impact in a time period and scenario.

Hydrologic basin	Red Sea-East Coast	Indus	Sabarmati	Ganges–Brahmaputra	Arabian Peninsula
Year	2100	2100	2100	2020	2100
Impact (Billions US 2020$)	−11086	−5447	−3008	−2405	−2357
Socioeconomics	SSP 5	SSP 3	SSP 5	SSP 4	SSP 5
Agriculture and land use	SSP 4	SSP 4	SSP 4	SSP 2	SSP 4
Other SSP elements linked to	Agriculture	Agriculture	Agriculture	Socioeconomics	Socioeconomics
Groundwater availability	25%	40%	50%	5%	5%
Reservoir storage	Restricted	Expanded	Restricted	Restricted	Restricted
ESM	NorESM	IPSL	GFDL	MIROC	MIROC
Land use scenario	UCT	UCT	UCT	FFICT	UCT

### Mitigation-scarcity trade-offs

**Fig. 3:**
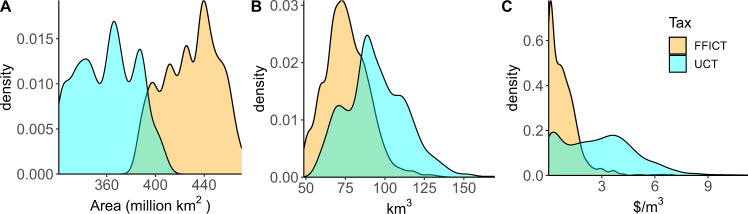
Land use scenario impacts. Density plots depicting the difference in tax regimes. The plot in **A** depicts the sum of global cropland over time under the two carbon tax regimes. The density plot in **B** shows water withdrawals in the Arabian Peninsula in FFICT (orange) and UCT (cyan) scenarios. The density plot in **C** depicts the shadow price of water in the Indus River basin in the two tax cases. Values in **B** and **C** are averaged over time. Total agricultural land increases under the FFICT while water price and water withdrawals increase under the UCT.

Pricing carbon emissions from the land-use sector often contributes to an economic tipping point because basins respond by intensifying agricultural land and increasing irrigation, thus exacerbating scarcity. When food demand increases, GCAM responds either by expanding agricultural land or intensifying existing agricultural land. With no price put on land-use change emissions (under the Fossil Fuel and Industrial Carbon Tax, or FFICT) it is more cost-effective to expand. Indeed, we find that scenarios with the FFICT use more agricultural land than the Universal Carbon Tax (UCT) scenarios (Fig. 3A). Conversely, the carbon prices under the UCT disincentivize expansion and therefore prompt intensification. Carbon prices are derived from the continued ambition scenario of the Nationally Determined Contributions in a future with medium challenges to adaptation and mitigation^40^ (see “Methods” section).

When intensification occurs, yields are increased by irrigating crops more instead of relying on rainwater. The intensity of agricultural land management also increases. These changes prompt greater water withdrawals (Fig. 3B). The shift from rainwater toward irrigated water also increases the price of water in the UCT scenarios (Fig. 3C). These results are especially significant in basins sensitive to land-use change. A previous study found that the FFICT prompts greater water withdrawals^41^. However, the study used a previous version of GCAM that did not have intensification options and assumed unlimited water. In that version, water use was proportional to land use. Therefore, when the UCT disincentivized expansion, water use was also limited. When extensification-intensification dynamics are considered, we find a substitution between water use and agricultural expansion. This finding emphasizes the importance of considering all trade-offs in mitigation policy options.

## Discussion

In this study, we use an economic surplus metric in order to quantify the economic impacts of water scarcity and the uncertainty of this impact due to different factors (i.e., population, agricultural productivity, etc.). Theoretically, basins would withdraw less when exposed to a limited supply of water and thus experience a negative economic impact, yet we find some basins capitalize on their water resources and become virtual water exporters in the face of global water scarcity. This dynamic would not be captured by looking at physical water scarcity metrics alone, nor by assessing economic impact at the basin-scale.

These variable responses to water scarcity are sometimes due to highly uncertain and largely uncontrollable factors such as the climate system. When normalized by a 2015 baseline, we find that uncertainty of economic impact due to Earth System Model forcing alone is often several thousand times higher than the uncertainty in the forcing itself (Fig. 2). Across the sampled states of the world, we find that slight deviations in precipitation drivers are almost always amplified as they propagate through markets. Since we have little control over uncertainty in the climate system, basin economies that are sensitive to fluctuations in hydro-climactic forcings will need especially robust water resource management frameworks in the future. Further, basins with the highest amount of impact variability due to climate uncertainty are often in politically unstable regions such as the Middle East. Thus, there is an even greater need to manage water resources in the most efficient way possible in the face of extreme uncertainty of economic impacts due to climate in these basins. Planners must also be aware of factors (e.g., population growth or carbon pricing regimes) that lead to economic tipping points in unstable basins.

Under the assumption that food production will always meet demand, implementing a Universal Carbon Tax prompts the intensification of agricultural land due to the increased cost of converting land for agricultural use. The intensification is enabled by increased irrigation and greater water withdrawals (Fig. 3). Thus, the effects of pricing carbon in a land-use policy on land intensification-extensification dynamics need to be taken into account in basins exhibiting high levels of water stress.

We find that most scenarios of interest (i.e., those that resulted in extremely high or low economic impact) are composed of a mix of SSP dimensions. This demonstrates the importance of using a scenario discovery framework in the context of a highly uncertain problem such as modeling water resources and the drawbacks of focusing on a limited set of narratives. In addition, the dimensions of high importance in certain basins are of less importance in others. Indeed, every dimension varied in this study was the most influential factor in determining the economic impact of water scarcity in at least one basin (Supplementary Fig. 12). Scenario discovery addresses this by identifying the most critical scenario components to the specific analysis context. There is no reason to expect universal shared scenarios will capture key challenges in each basin (or indeed in any), and it is very difficult to anticipate what combinations of factors present challenges in every basin before doing extensive exploration. Scenario discovery is a promising approach to identify relevant scenarios to inform water scarcity analyses. In addition, while this work assessed the economic impact in water markets alone, future work could make use of a Computable General Equilibrium model where the interactions between all markets would be accounted for (see “Methods” section). Indeed, we hope this work provides the basis for similar analyses across a range of hydro-economic models to ascertain the sensitivity of our results to model structure. Confidence in our metric depends on the fidelity of the selected hydro-economic model, so future work would benefit from expanded data collection of socio-technological drivers of regional and sectoral water consumption to improve those underlying models. This study’s use of a coupled partial equilibrium-hydrologic model to perform an extensive uncertainty analysis is novel to the integrated assessment modeling literature and enables the discovery of important multi-scale dynamics such as a basin’s wide range of adaptive responses to water scarcity.

## Methods

### Human-earth system model

Multiple factors affect water demand including population, wealth abundance and distribution, agricultural technology and practices, technological improvements, and carbon and land-use policy. These factors all interact with each other and with the climate system. It is therefore necessary to use a model that includes detailed representations of these systems and the interdependent endogenous choices that shape them. To this end, we have used a partial equilibrium model in order to represent the affected systems with as much detail as possible.

This study makes use of the Global Change Analysis Model (GCAM), a human-Earth system model that has been used by numerous agencies to make informed policy decisions^36^. GCAM is a complex model that decomposes the world into 32 geopolitical regions, 384 land-use regions, and 235 water basins^36^. GCAM includes coupled representations of the Earth’s climate, economic, hydrologic, land-use, and energy systems. These systems are expressed in varying degrees of detail. Population and GDP growth are represented as simple exogenous model inputs. Energy and land-use systems are represented in more detail, with shares of supplies and technologies competing using a logit model^36^. Renewable technologies within the model become more efficient over time and therefore some processes such as solar energy production become more competitive. Nonrenewable resources such as oil and fossil groundwater are modeled with graded supply curves and become more expensive as the levels are used up over time. Shares of energy production technologies may change based on different policy choices. For example, a carbon tax may increase the feasibility of using renewable energy sources. These policies may also impact the shares of land uses (e.g., the carbon tax may prompt afforestation).

### Water demand and supply

GCAM allows users to specify water constraints and to link water supply to Xanthos, an extensible hydrologic model^42^. Previous versions of GCAM have introduced the water system but have limited its capabilities to computing water demands. The current system calculates both supply and demand and balances the two quantities by solving for an equilibrium regional shadow price for water^38,43,44^. Water demand in GCAM is modeled through six sectors: irrigation, livestock, municipal, manufacturing, primary energy, and electricity generation^25^. Irrigation demand is based on biophysical water demand estimates for twelve crop classes^25^. Water demand for irrigation is determined by deducting green water (i.e., water available for use by plants) on irrigated areas and green water on rain-fed areas from total water consumption. Livestock water demand is computed using the consumptive rates for six livestock types (cattle, buffalo, sheep, goats, pigs, and poultry) and estimates of livestock density in 2000^25^. Water withdrawals for electricity generation are related to the amount of electricity generated in each region. Once-through cooling systems compete with evaporative cooling systems with the latter becoming more prevalent over time^25^. Water use in the primary energy sector (i.e., the water used to extract natural resources) is calculated using estimates of energy production in each region along with water use coefficients. Municipal water demand is modeled using population, GDP, and assumptions about technological efficiencies^36,41^. Finally, manufacturing water demand is the total industrial water withdrawals less the energy-sector water withdrawals^25^. Consumption is calculated using exogenous consumption to withdrawal ratios for industrial manufacturing^25^.

Water supply in GCAM is modeled using three sources: surface water and renewable groundwater, nonrenewable groundwater, and desalinated seawater. Similar to technology use within GCAM, these sources of water compete using a logit structure based on price. Surface water is typically used first in larger quantities than its competing sources as it is the cheapest source of water. The upper limit of surface water in a basin is taken to be the mean average flow modeled using Xanthos, which calculates water supply at a monthly time step using evapotranspiration, water balance, and routing modules^42^. Accessible water^38^ is assumed to be the volume of runoff available even in dry years in addition to reservoir storage capacity (after removing environmental flow requirements). The estimates of accessible water and basin runoff are used as inputs in GCAM. After the renewable water supply is fully consumed, GCAM will either use desalinated water or nonrenewable groundwater depending on the relative shares computed in the price-based logit structure^38^. Nonrenewable groundwater increases in price as more of the resource is consumed. The groundwater supply curves account for geophysical characteristics such as aquifer thickness and porosity, as well as economic factors such as the cost of installing and operating the well. As the price of extraction rises, desalination becomes more competitive, resulting in wider use of desalinated water^44^.

Basin-specific water policies are not represented within GCAM or indeed any global model. The level of detail needed to represent existing water markets and policies exceeds the capabilities of a global model. GCAM does, however, enforce a subsidy on water for agricultural sectors^36^. Imposing this subsidy in GCAM’s water markets allows water to be allocated first to agricultural producers. This behavior mimics the effect of traditional water rights in that senior rights are usually given to agricultural producers. The water markets within GCAM operate by generating a “shadow” price of water, which reflects the economic value of the last unit of water in terms of the water’s contribution to production. When water supply becomes a binding constraint in a particular water basin, the shadow price of water rises because users cannot use more water than there is in the basin. This forces a reduction in the production of the goods and services that rely on water as an input. Clearly, this approach is a simplification, but it marks an improvement over what is most often done where the implications of water scarcity are ignored (i.e., direct and indirect feedbacks associated with unsatisfied water demands are not captured, and analyses are limited to how water scarcity may increase or decrease in the future without a mechanism for dynamic adaptation measures).

We compute the difference in total economic surplus in these simplified water markets (i.e., the sum of producer surplus and consumer surplus) between a control scenario with no water constraints and its paired limited water scenario (see next section).

Capturing economic impact in the entire economy would require a general equilibrium model. However, general equilibrium models necessarily lose some detail in sectoral resolution so that they can capture market interactions. Water is a non-substitutable input to most markets in the human system and so most market interactions will be represented by the changes in water markets when conditions are perturbed. The surplus change in the water markets includes both direct effects (e.g., restricted supply) and indirect effects (e.g., demand shifts in adjacent markets). There may be economic effects not captured by looking at water markets alone, which could be investigated in future work that employs a computable general equilibrium model. Numerous previous studies have assessed economic impact in water markets using both types of models^45^.

### Scenario design

We utilize a scenario discovery approach^35^ to study the uncertainty in physical water scarcity and its economic impacts. Using this approach, scenarios are generated using all possible combinations of discrete levels of uncertain factors. All scenarios are weighted equally during scenario exploration so as not to presume the likelihood of outcomes a priori. Doing so may leave the system vulnerable to unanticipated events. In addition, in complex adaptive systems such as the human-Earth system, the main drivers of an outcome of interest may be non-intuitive and context-specific^34^. The traditional “predict-then-act” approaches^46^ to planning implies a more complete understanding of the system and of future circumstances than is often the case, which can, in turn, lead to myopic decisions^35^. Alternatively, scenario discovery gives equal weight to all possible future system trajectories (i.e., population, wealth, energy prices) and finds the most influential factors driving outcomes of interest-based on the results of all scenarios. Planners can then make robust management decisions based on the influential factors and their uncertainties as opposed to designing based on a few future projections.

In this study, we use scenario discovery to determine the relative influence of seven dimensions in driving highly consequential economic outcomes due to water scarcity (Supplementary Fig. 2). These factors include socioeconomic conditions (*S*), agricultural yield assumptions (*G*), groundwater supply (*W*) and reservoir storage (*R*) levels, climate trajectories (*E*), and land-use scenarios (*T*). All factors are represented in Eq. (1) and are discussed in more detail below. Every scenario *n* is composed of a distinct combination of the levels of each factor.

1 $$n_{sglwret} \in \left[ {\left( {s \in S} \right) * \left( {g \in G} \right) * \left( {l \in L} \right) * \left( {w \in W} \right) * \left( {r \in R} \right) * \left( {e \in E} \right) * \left( {t \in T} \right)} \right]$$

Settings for the first three dimensions are taken from GCAM’s implementation of the Shared Socioeconomic Pathways (SSPs)^47–49^. The SSPs are based on plausible but distinct narratives that envision how the century will unfold^47^. The five SSPs correspond to the four combinations of high and low challenges to adaptation and mitigation of climate change with a fifth narrative that lies in the middle of the adaptation-mitigation challenge plane. The implementations of the SSPs within GCAM are made up of factors including population and GDP, agricultural yields, carbon sequestration implementation, renewable energy use, fossil fuel extraction cost, and energy demand^48^. This study included the population/GDP component and the agricultural component of the SSPs. The remaining components of the SSP framework were linked to either the population/GDP or agricultural component. For instance, in one scenario, SSP 3 fossil fuel extraction costs and renewable energy assumptions would be present with SSP 3 socioeconomics and SSP 5 agriculture assumptions; the converse scenario of this dimension would include SSP 5 fossil fuel extraction costs, renewable energy assumptions, and agriculture yields and SSP 3 socioeconomics. This switch (*L*) represents the third dimension of the design. Previous work found the socioeconomic and agricultural and land use elements of the SSPs had the most profound impact on water use^34^, thus we linked the other elements to ensure the scenario design emphasized potentially impactful factors.

The next three dimensions relate directly to the water supply. Groundwater availability is constrained at different levels (5%, 25%, and 40% of the physical water availability) that reflect the economic feasibility of extracting groundwater using the methodology within Turner et al. (2019a)^50^. We also vary reservoir storage estimates using two extremes following the methodology in Turner et al. (2019b)^44^. A restricted scenario indicates that reservoir storage remains constant from the present to the end of the century while an expanded scenario expresses a linear increase from current levels to maximum storage capacity (meaning all accessible water is stored) at the end of the century^44^. The Earth System Model forcing trajectories used as input to Xanthos were also varied between GFDL, MIROC, IPSL, HadGEM2, and NorESM^51–55^.

The final dimension corresponds to land-use scenarios formed by mitigation policies. The first, a Universal Carbon Tax (UCT) scenario, imposes a carbon tax on all sectors of the economy including emissions from land-use change. This scenario has many different land-use implications than the alternative scenario that employs the Fossil Fuel and Industrial Carbon Tax (FFICT) which does not price changes in land use (e.g., preserving grasslands and forests rather than expanding agriculture). To construct these scenarios, we use a carbon price trajectory that approximates the continued ambition scenario of the Nationally Determined Contributions (NDCs) as implemented in Fawcett (2015) and revised in Cui et al. (2018)^40,43^. This scenario assumes that countries continue decarbonization at the same rate as was necessary to meet the NDCs by 2030. The price of carbon at the reference scenario (SSP 2) for the continued ambition trajectory was used globally for all scenarios. The price begins at $21/ton of CO2 and increases to $233/ton of CO2 by the end of the century. These carbon prices are applied to all sectors (under the UCT) or to every sector but land-use change (under the FFICT).

In total, all unique combinations of the levels of these dimensions (i.e., the size of the set in Eq. 1) yield 3000 scenarios. Of the 3000, the total surplus could be calculated for 2876 scenarios without integration errors. Importantly, using a single carbon price trajectory while varying other socioeconomic and climatological factors yields a spread of emission trajectories. This will produce inconsistencies in a given scenario to the extent inputs depend on exogenous forcing trajectories. In this study, this is most important to the generation of hydrologic realizations (to compute available renewable water), where the Xanthos model was forced using several downscaled ESM simulations of RCP 4.5 even though the actual forcing trajectories varied across scenarios. Since climate change will impact the water cycle^56^, the amount of renewable water would also be different in each scenario had Xanthos been run endogenously. However, the magnitude of this difference is highly uncertain, as climate models have been found to cause as much or more uncertainty in hydrologic realizations as the RCPs themselves^57^. Thus, it is not clear that imposing an emissions cap to ensure consistency in forcing would better characterize hydrologic uncertainty. Future studies, for instance, those focused on the cost of meeting mitigation targets, may instead choose to vary prices rather than emissions, but this is beyond the scope of this work.

In addition to the dimensional components of the design, we added further inputs to reflect the recent advances of GCAM. Agricultural yield inputs based on Earth System Model, Representative Concentration Pathway (RCP)^58^ and SSP were included, as well as hydropower inputs based on SSP, RCP, and ESM, and technological water demand estimates based on SSP^49,59^.

### Water scarcity metrics

Many different metrics for measuring water scarcity have been proposed^56,60,61^. The most commonly used metrics typically compute physical water scarcity and exclude the socioeconomic information necessary to understand adaptive capacity. For example, the Water-To-Availability ratio (WTA) is computed as water withdrawals over renewable water supply^14,25^. Several holistic metrics exist that include socioeconomic information such as the Human Development Index^62^, though these metrics face the challenge of subjectively determining how to weight socioeconomic indicators relative to one another^60^.

This study examines water scarcity vulnerability using a metric that accounts for the economic impact of water scarcity within a hydrologic basin. We use the change in economic surplus in water markets between a basin with unlimited water and one with physical constraints on the water supply to calculate this economic impact. This difference consists of the direct impacts of changes in the water supply, as well as the indirect impacts from markets that rely on water. From this point on, we will refer to the surplus change in water markets as simply the surplus change, or economic impact.

Change in economic surplus has been used in many disciplines since its inception^63^. It has been used to assess the impact of climate change on agriculture^64,65^, as well as potential infrastructure projects^66^ and adaptation policies^67,68^. Its continued use is due in part to its ease of implementation, its theoretical simplicity, and its ability to capture changes across sectors. These qualities are highly beneficial in a water scarcity metric. Computing the loss of surplus due to some factor requires a counterfactual scenario in which that factor is absent. This presents a problem when applying this type of metric to any historical data, including water scarcity: water scarcity has always been present. Even a synthetic history with unlimited water would be inadequate as all other historical values depend on historical water scarcity levels. Still, our metric has significant advantages over conventional physical water metrics that lack information about the ability of the basin to respond to water stress.

Here economic impact is defined as:

2 $$I = T_{{\mathrm{constrained}}} - T_{{\mathrm{unlimited}}}$$

where *T* represents the total economic surplus (Supplementary Fig. 1). In this study, the economic impact is reported using its log-modulus and has units billions of 2020 US dollars:

3 $$L = {\mathrm{sign}}\left( I \right) * \log \left[ {\left| I \right| + 1} \right].$$

Thus, an impact value of −2 would correspond to a loss of 100 billion 1975 US dollars or 2.3% of US GDP in 2018 after adjusting for inflation^69^.

Sign changes in economic impact correspond to shifts in water demand in a basin between unlimited and limited water scenarios. If the total surplus gained from increased withdrawals exceeds the consumer surplus lost by low-demand consumers when water limitations are imposed, basins experience a positive impact. This counter-intuitive case could result when basins become virtual water exporters when global physical constraints are imposed. With water constraints in place, such regions now have a comparative advantage in producing water-intensive goods (notably agricultural products, see Supplementary Fig. 3); therefore, they capture greater market share in the water-constrained scenarios. The increased production of these goods translates into a positive shift in demand in water markets. The additional economic activity also increases the value of water as consumers’ willingness to pay for goods increases. This additional economic activity manifests as a larger economic surplus, which translates to a more positive impact. The magnitude of the metric gives an indication of the difficulty of overcoming water scarcity within a basin since the economic impact depends on the value put on water. Higher values of water correspond to higher magnitudes of economic impact.

### CART

To uncover the most influential factors that lead a basin to experience positive versus negative impact, we used the Classification and Regression Trees (CART) algorithm^70^. The CART algorithm has been found useful in determining important factors and scenarios of interest in previous studies^34,35^. CART operates by performing binary splits of the data to create the purest possible subgroups. In this study, we use CART to identify the factors that lead to the worst-case scenarios with respect to the economic impact metric. Examining this continuous metric necessitates the use of the regression approach of CART. The regression approach uses an Analysis of Variance (ANOVA) method to discover the purest subgroups. Splits work to maximize the variance between groups and minimize variance within groups.

## Supplementary information

Supplementary Information

## Data Availability

Requests for raw data should be made to flannery.dolan@tufts.edu. Processed data is available at 10.5281/zenodo.4470017^71^.

## Code availability

Code to generate the main text figures and calculate economic impact can be found at 10.5281/zenodo.4470017^71^.
