# Supplementary material for: Evaluating the economic impact of water scarcity in a changing world
Source: Nat Commun. 2021 Mar 26;12:1915. doi: 10.1038/s41467-021-22194-0 (PMC7997906; doi:10.1038/s41467-021-22194-0)
Supplement: Supplementary file 1 — Supplementary Information [file 41467_2021_22194_MOESM1_ESM.pdf]

Supplementary Information

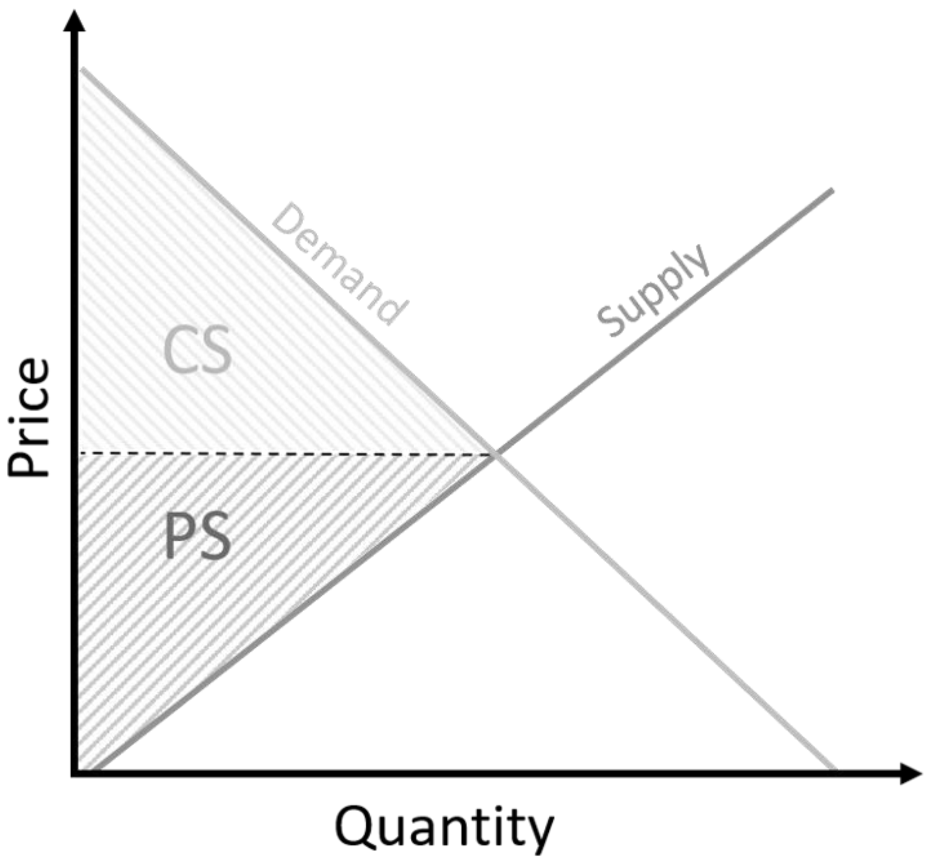

Supplementary Figure 1 Economic surplus: Consumer surplus (CS) and producer surplus (PS) are differentiated by the equilibrium price of a good (the price at which supply intersects with demand). The sum of these areas is the total surplus and has units of dollars.

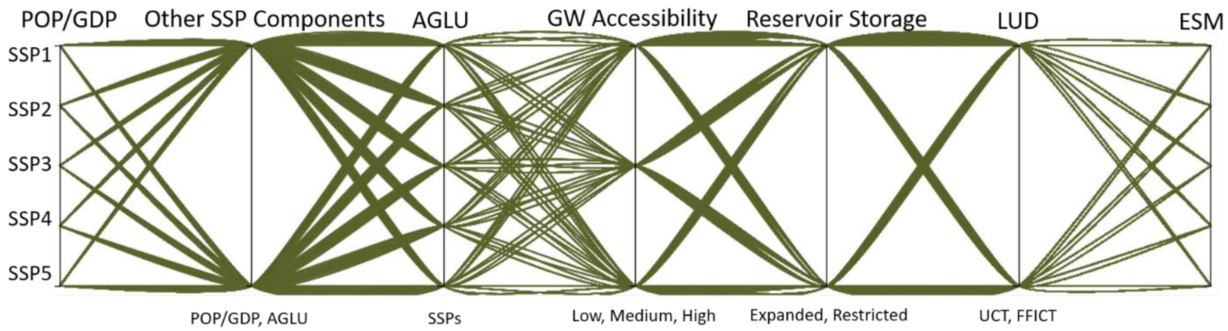

Supplementary Figure 2 Experimental design: The factorial sampling of the experimental design.

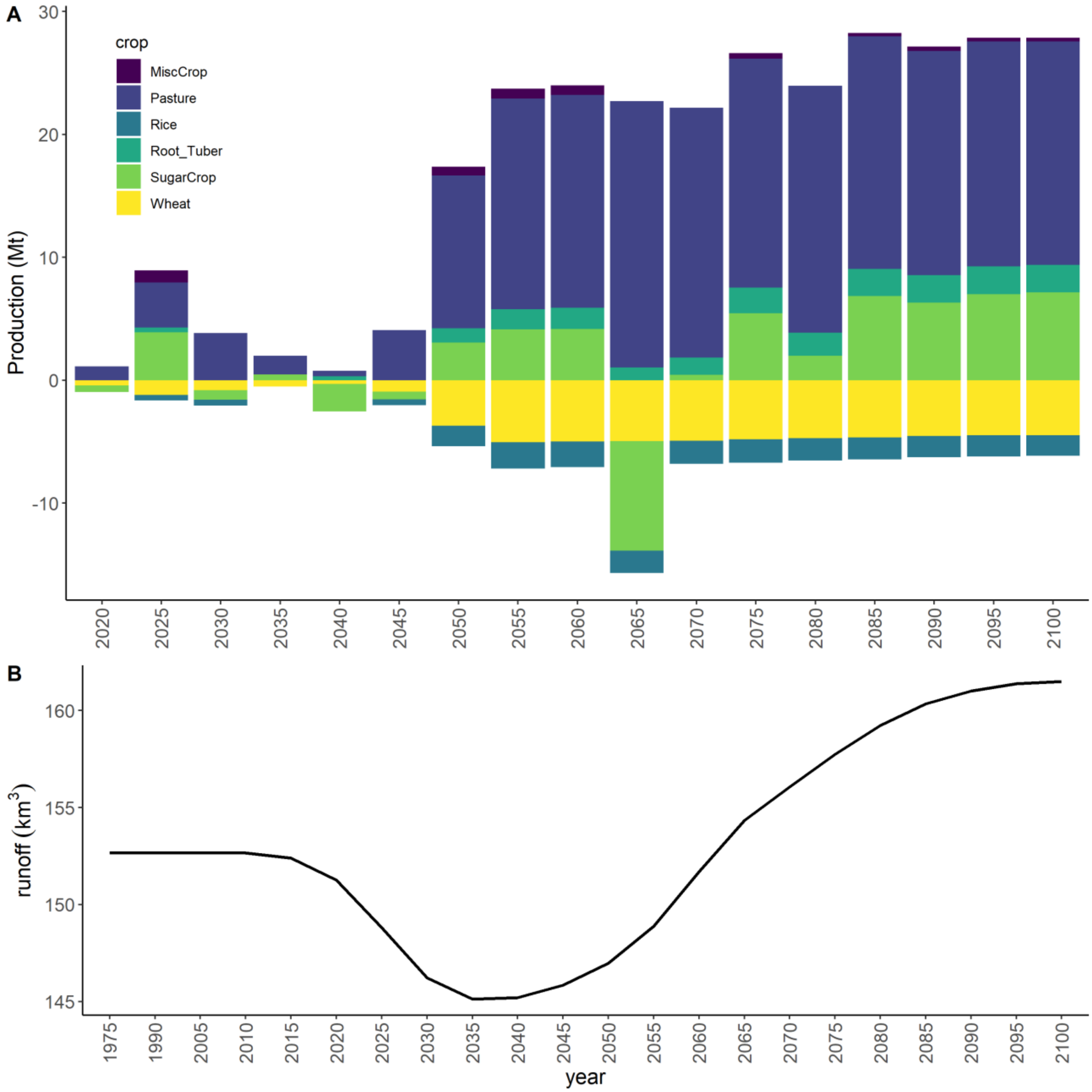

Supplementary Figure 3 Agricultural production-runoff relationship: The difference in agricultural production between the constrained water scenario and the unlimited water scenario. A) This scenario pair has SSP 5 socioeconomics, SSP 4 agriculture, SSP 4 assumptions in the other dimensions and the UCT. The constrained scenario has low groundwater availability, expanded reservoir storage and is forced with NorESM. This scenario has the highest positive impact over time in the ensemble in the Indus Basin. Increases in pasture are due to increased dairy production. The runoff is plotted in B).

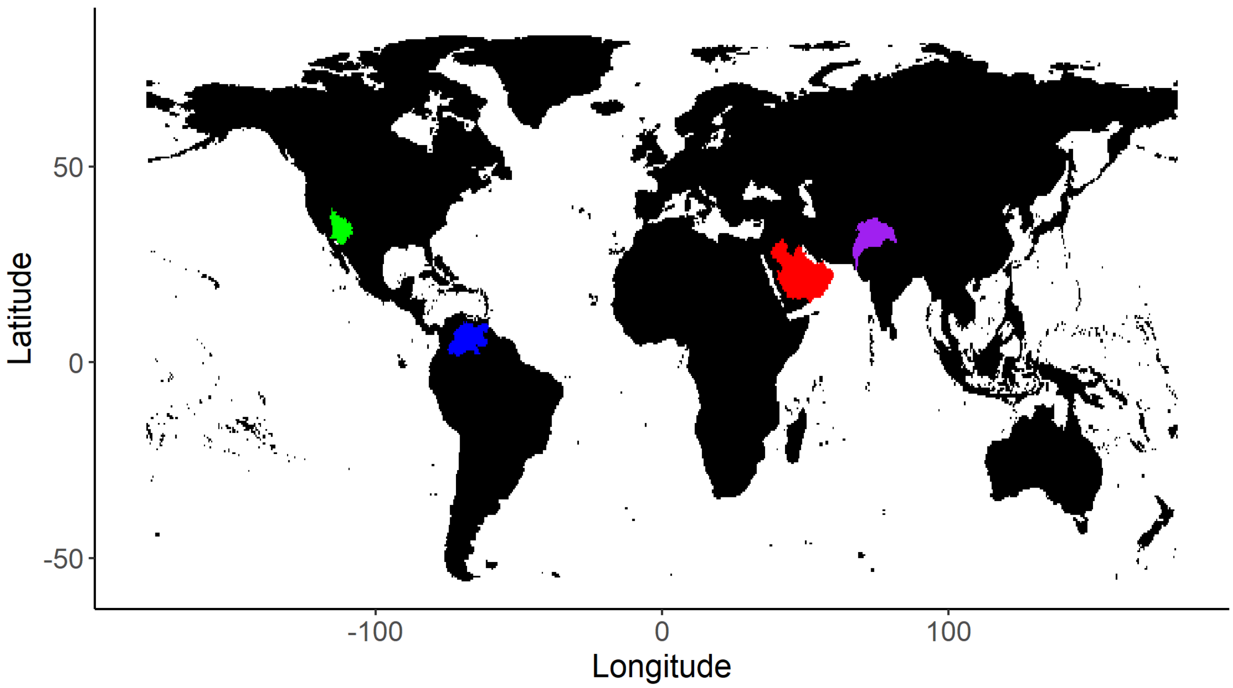

Supplementary Figure 4 Location of highlighted basins: The basins highlighted in this study. The Lower Colorado River Basin (green), the Orinoco Basin (blue), the Arabian Peninsula (red), and the Indus Basin (purple).

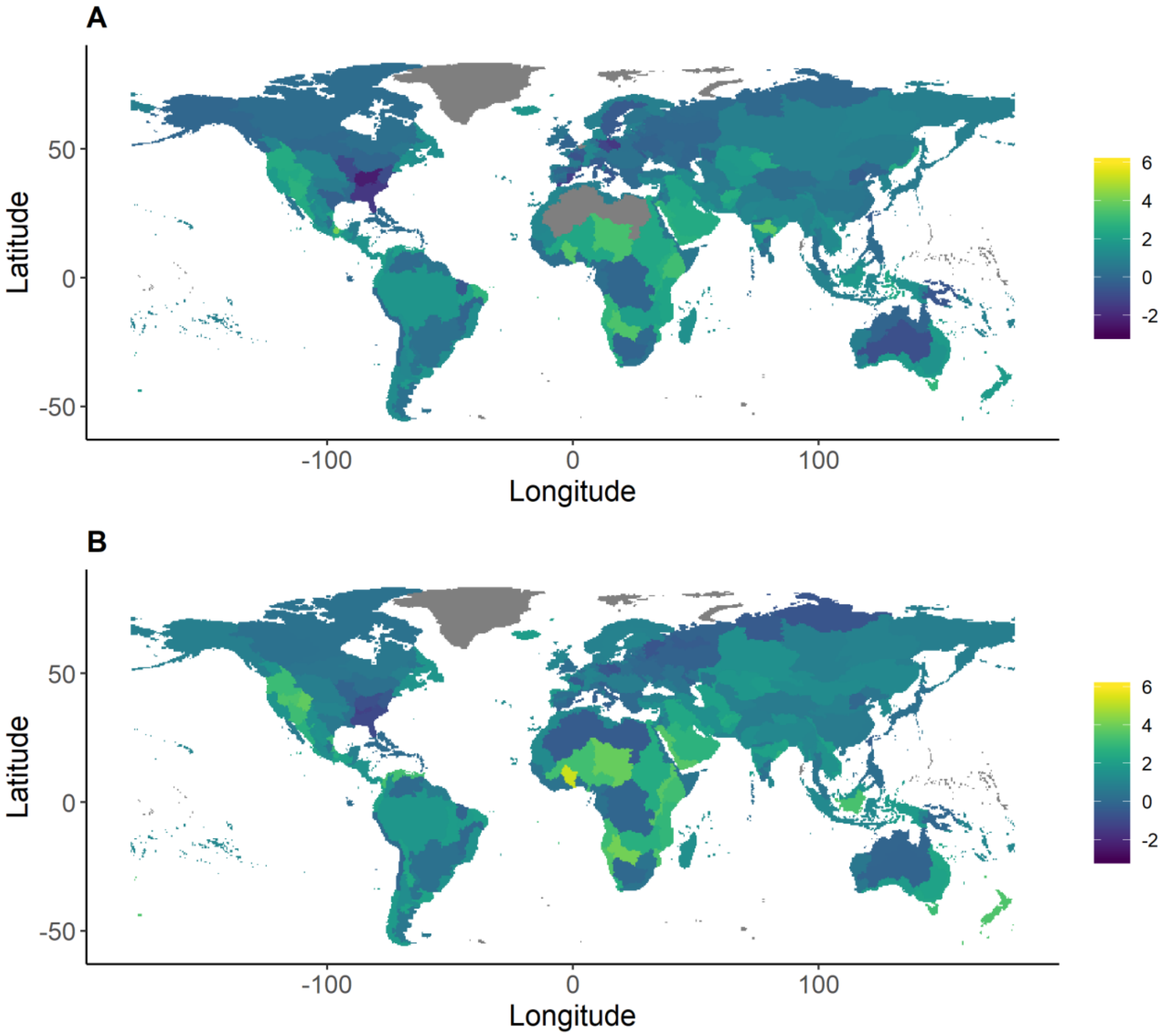

Supplementary Figure 5 Climate uncertainty amplification ratios: Log transforms of the maximum climate uncertainty amplification ratios in each basin in a single scenario with SSP 1 (A) and SSP 5 (B) demand assumptions and low water supply assumptions. Higher amplification ratios arise in higher demand scenarios.

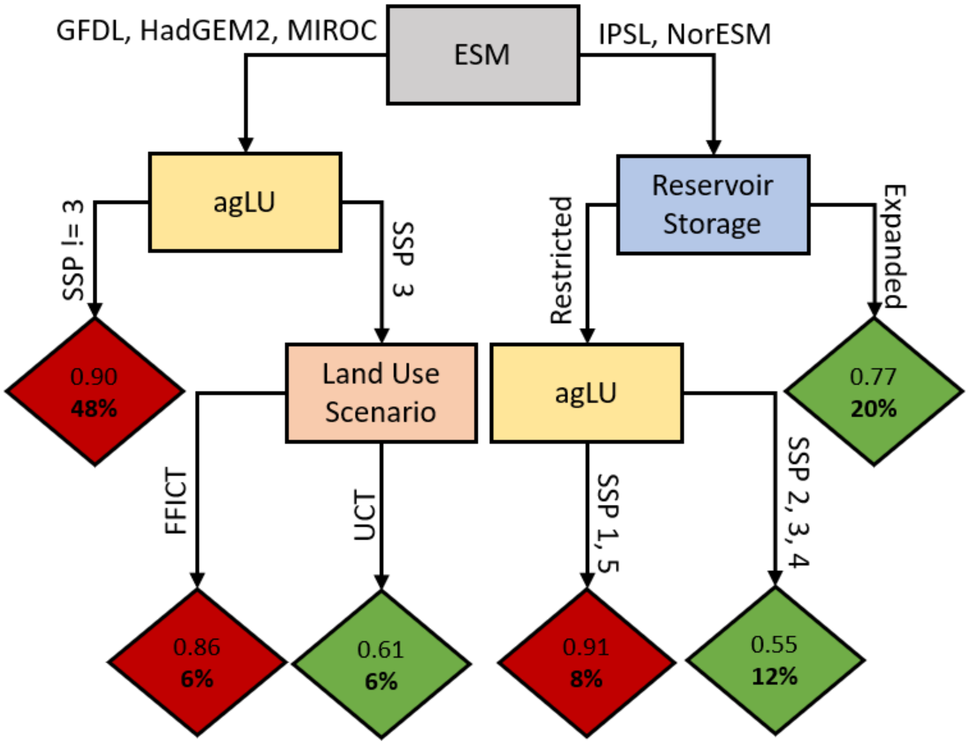

Supplementary Figure 6 Arabian Peninsula classification tree of sign of economic impact: CART classification of positive versus negative economic impact summed over time in the Arabian Peninsula. Red end nodes represent negative impact subgroups and green end nodes represent positive impact subgroups. The fraction at the top of each node shows the purity of each node. For instance, a red node with 0.91 shows that 91% of scenarios in that subgroup are negative. The percentage in each end node is the percent of scenarios out of the total within that subgroup. Earth System Model forcing is the most influential factor in determining if impact is positive or negative.

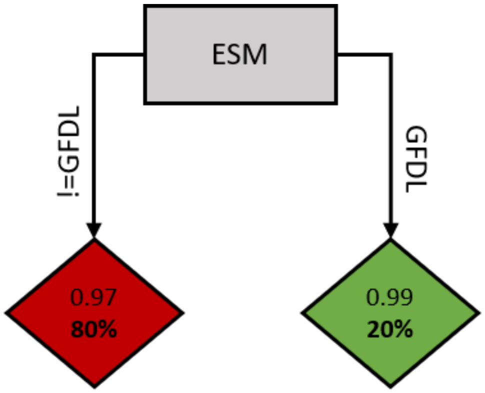

Supplementary Figure 7 Lower Colorado River classification tree of sign of economic impact: CART classification of positive versus negative economic impact summed over time in the Lower CO River Basin. Red end nodes represent negative impact subgroups and green end nodes represent positive impact subgroups. The fraction at the top of each node shows the purity of each node. For instance, a red node with 0.97 shows that 97% of scenarios in that subgroup are negative. The percentage in each end node is the percent of scenarios out of the total within that subgroup. Earth System Model forcing is the most influential factor in determining if impact is positive or negative.

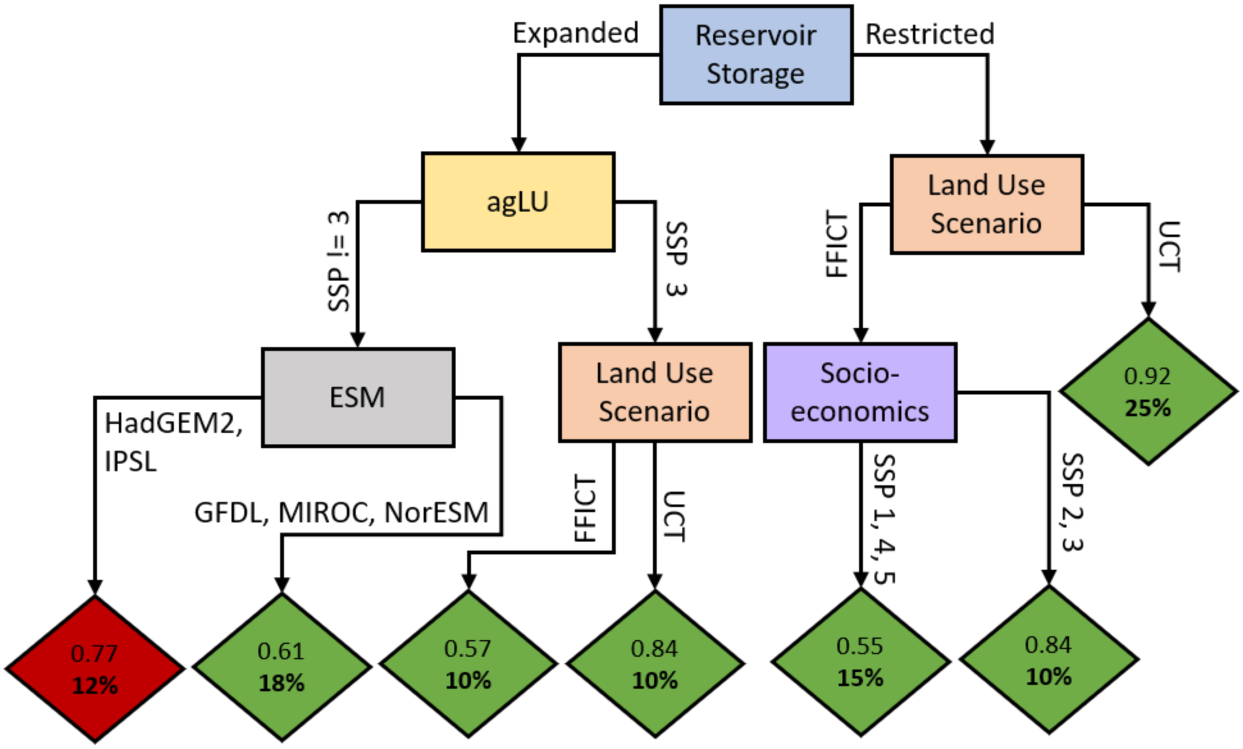

Supplementary Figure 8 Orinoco classification tree of sign of economic impact: CART classification of positive versus negative economic impact summed over time in the Orinoco Basin. Red end nodes represent negative impact subgroups and green end nodes represent positive impact subgroups. The fraction at the top of each node shows the purity of each node. For instance, a red node with 0.77 shows that 77% of scenarios in that subgroup are negative. The percentage in each end node is the percent of scenarios out of the total within that subgroup. Earth System Model forcing is one of the most influential factor in determining if impact is positive or negative.

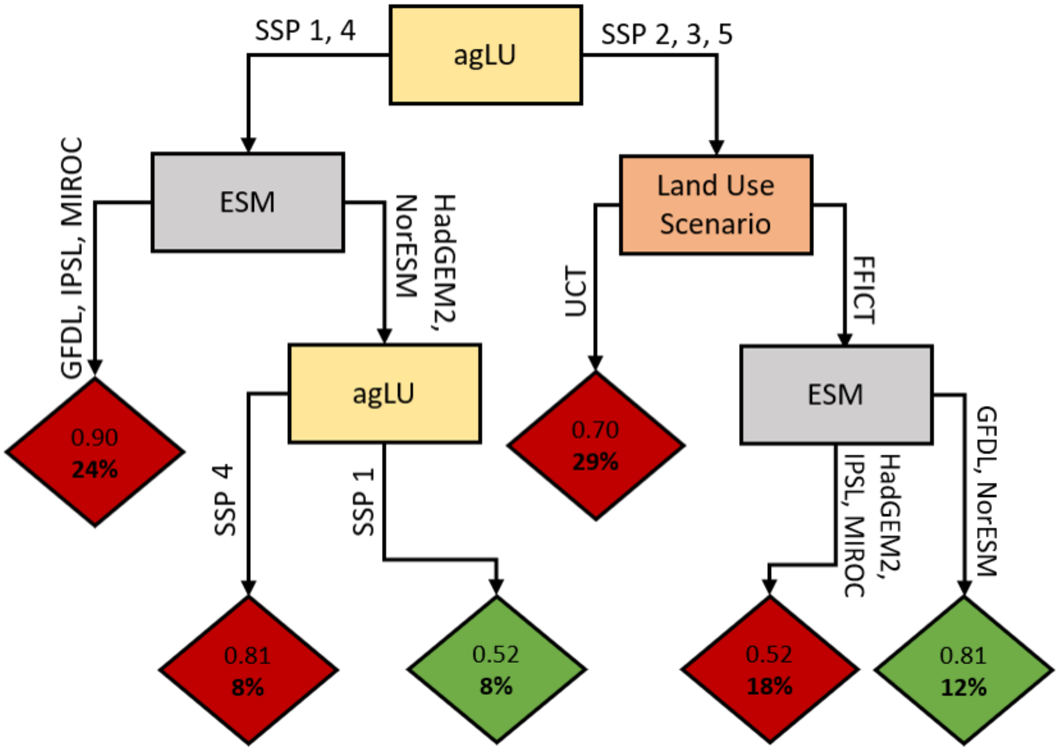

Supplementary Figure 9 Indus classification tree of sign of economic impact: CART classification of positive versus negative economic impact summed over time in the Indus Basin. Red end nodes represent negative impact subgroups and green end nodes represent positive impact subgroups. The fraction at the top of each node shows the purity of each node. For instance, a red node with 0.81 shows that 81% of scenarios in that subgroup are negative. The percentage in each end node is the percent of scenarios out of the total within that subgroup. Earth System Model forcing is one of the most influential factor in determining if impact is positive or negative.

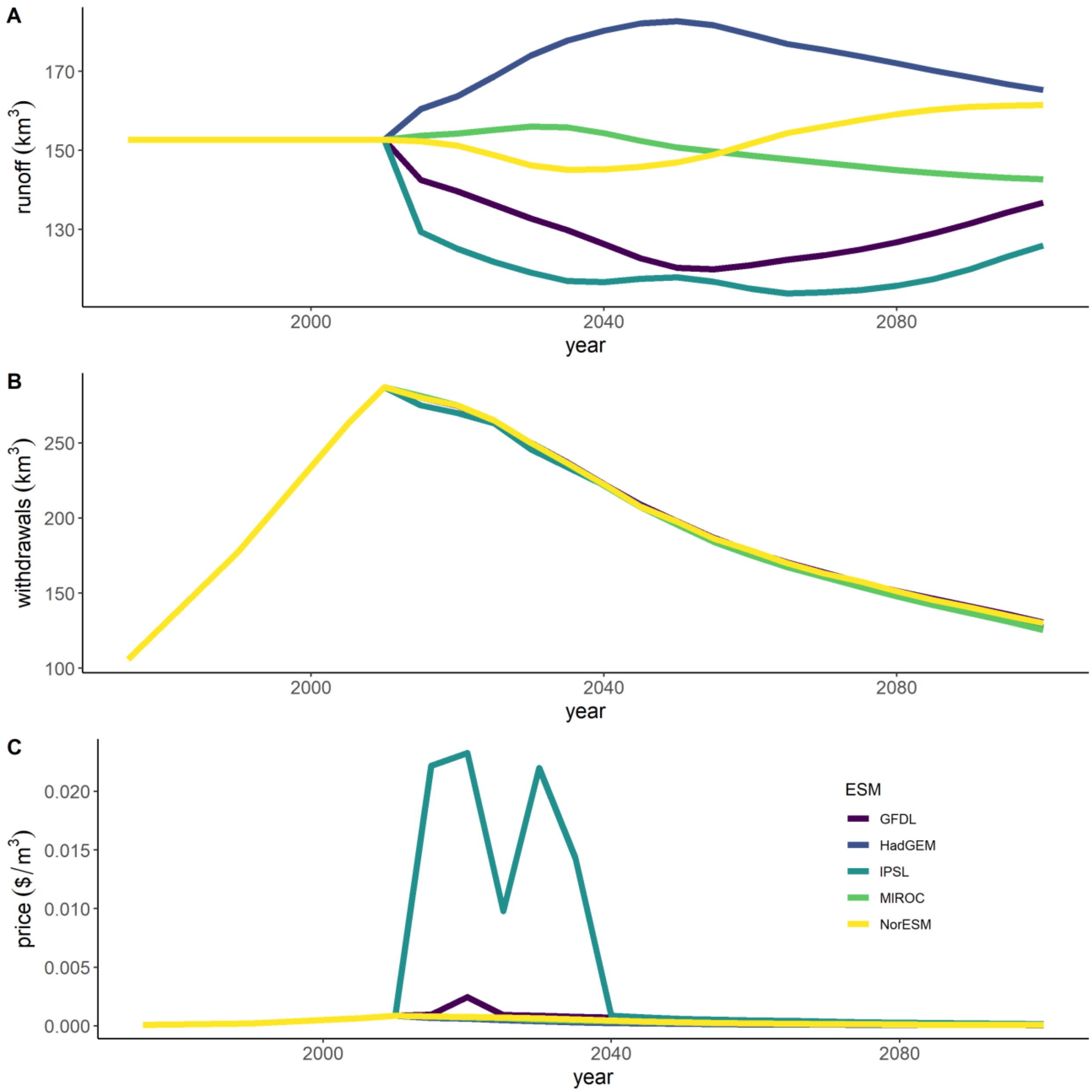

Supplementary Figure 10 Runoff-water price relationship: Runoff A), withdrawals B), and water price C) plotted for each ESM in the Indus Basin. The group of scenarios plotted corresponds to those in Figure 2 B. Changes in supply coupled with static demand leads to variations in the price of water.

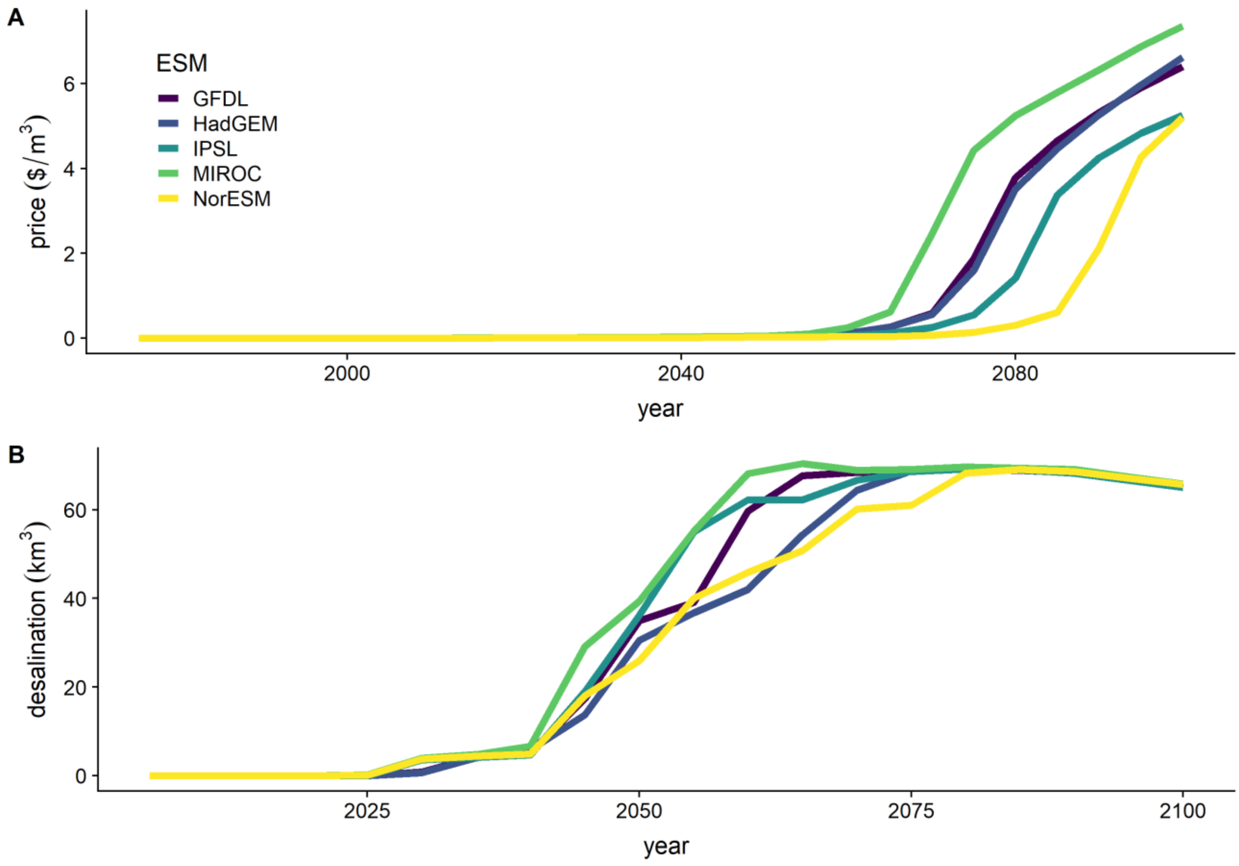

Supplementary Figure 11 Desalination-water price relationship: Water price A) and desalination production B) plotted for each ESM in the Arabian Peninsula. The group of scenarios plotted corresponds to those in Figure 2 E. The price of water increases as alternative sources of water, such as desalination, are used.

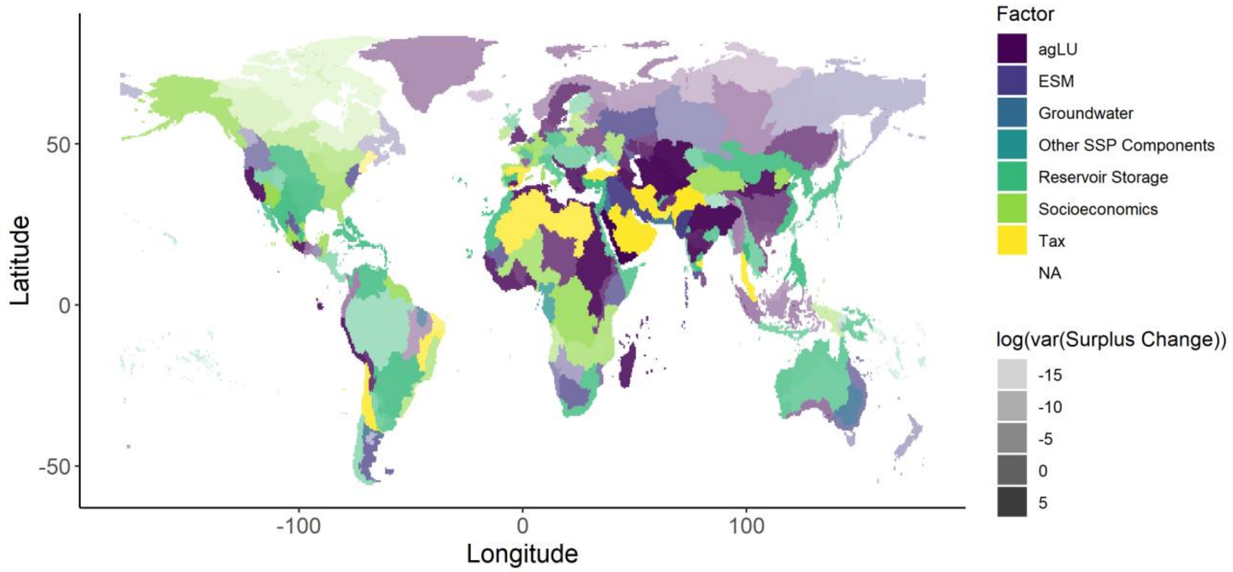

Supplementary Figure 12 First node of regression tree of economic impact: The first factor in a CART regression of the economic impact of water scarcity summed over time. All dimensions in the experimental design are the most influential factor in determining economic impact in some basin. Basins are shaded according to the magnitude of their variance.

Hydrologic Basin	Percent Positive Scenarios
Adriatic Sea-Greece-Black Sea Coast	82.1
Africa-East Central Coast	58.7
Africa-Indian Ocean Coast	70.7
Africa-North Interior	89.7
Africa-North West Coast	85.4
Africa-Red Sea-Gulf of Aden Coast	49
Africa-South Interior	23
Africa-West Coast	78.9
Amazon	94.4
Amu Darya	27.9
Amur	85.1
Angola-Coast	66.6
Arabian Peninsula	30.6
Arabian Sea Coast	62.4
Arctic Ocean Islands	95
Arkansas White Red	91.6
Atlantic Ocean Seaboard	95.7
Australia-East Coast	83.9

Hydrologic Basin	Percent Positive Scenarios
Australia-Interior	94
Australia-North Coast	92.2
Australia-South Coast	81.4
Australia-West Coast	75.8
Baja California	63.3
Baltic Sea Coast	87.4
Bay of Bengal-North East Coast	1.9
Black Sea-North Coast	92.7
Black Sea-South Coast	77.4
Bo Hai-Korean Bay-North Coast	82.4
Brahamani	0.3
California River	76.7
Caribbean	89.9
Caribbean Coast	86.5
Caspian Sea Coast	83.7
Caspian Sea-East Coast	52.6
Caspian Sea-South West Coast	54.3
Cauvery	19.5
Central Iran	31.7
Central Patagonia Highlands	56.2
Chao Phraya	16.1
China Coast	89.4
Churchill	34.8
Colombia-Ecuador-Pacific Coast	36.6
Congo	33.8
Danube	35.9
Daugava	93.6
Dead Sea	43.3
Denmark-Germany Coast	59.8
Dnieper	93.9
Dniester	92
Don	78.4
Douro	56.3
East Brazil-South Atlantic Coast	50
Eastern Jordan-Syria	24.3
Ebro	88.2
Elbe	93.6
Ems-Weser	63.7
England and Wales	68

Hydrologic Basin	Percent Positive Scenarios
Farahrud	64.9
Finland	59.1
Fly	18.3
France-South Coast	89.4
France-West Coast	59.4
Fraser	96
Ganges-Bramaputra	77.1
Gironde	79.9
Gobi Interior	87.7
Godavari	39.9
Great	84.2
Great Lakes	55.7
Grijalva-Usumacinta	30.3
Guadalquivir	35.1
Guadiana	25.2
Gulf of Guinea	35.7
Gulf of Thailand Coast	92.5
Hainan	68.7
Hamun-i-Mashkel	57.7
Hawaii	45
Helmand	74.8
Hong-Red River	35.7
Huang He	82.6
Hudson Bay Coast	46.8
Iceland	78.4
India East Coast	17.8
India North East Coast	18
India South Coast	38.3
India West Coast	0.3
Indus	33.9
Ireland	58.7
Irian Jaya Coast	92.1
Irrawaddy	56.4
Isthmus of Tehuantepec	73.8
Italy-East Coast	92.3
Italy-West Coast	92.9
Japan	65.7
Java-Timor	83.1
Kalimantan	92.7

Hydrologic Basin	Percent Positive Scenarios
Kara Sea Coast	92.3
Krishna	42.2
La Plata	94.5
La Puna Region	65.2
Lake Balkash	79.6
Lake Chad	23.5
Lena	40.4
Limpopo	79.7
Loire	90.2
Lower Colorado River	21.5
Lower Mississippi River	79.9
Mackenzie	32.6
Madasgacar	90.9
Magdalena	64.9
Mahandi	0.3
Mahi	25.1
Mar Chiquita	94.9
Mediterranean Sea Islands	87.9
Mediterranean Sea-East Coast	30.6
Mediterranean South Coast	79.3
Mekong	86.9
Mexico-Interior	73.1
Mexico-Northwest Coast	39.3
Mid Atlantic	89
Missouri River	88.7
Murray-Darling	49.5
Namibia-Coast	68.7
Narmada	79.9
Narva	88.3
Negro	83.8
Neman	91.9
Neva	87.6
New England	90.5
New Zealand	43.6
Niger	23.2
Nile	91.7
North and South Korea	91.7
North Argentina-South Atlantic Coast	73.2
North Borneo Coast	47.6

Hydrologic Basin	Percent Positive Scenarios
North Brazil-South Atlantic Coast	93.9
North Chile-Pacific Coast	92
North Gulf	33.3
Northeast South America-South Atlantic Coast	82.8
Northern Dvina	92.3
Northwest Territories	33.8
Ob	61.1
Oder	95
Ohio River	91
Orange	85.7
Orinoco	62.3
Pacific and Arctic Coast	95
Pacific Central Coast	58.1
Pacific Northwest	76.8
Palau and East Indonesia	93
Pampas Region	84.8
Papaloapan	62.5
Papua New Guinea Coast	83.6
Parnaiba	68.6
Peninsula Malaysia	89.5
Pennar	14.6
Persian Gulf Coast	56.3
Peru-Pacific Coast	57.8
Philippines	94.2
Plateau of Tibet Interior	33.8
Po	91.9
Poland Coast	91.5
Red Sea-East Coast	1.5
Rhine	64
Rhone	61.3
Rift Valley	43.1
Rio Balsas	64.1
Rio Grande River	54
Rio Lerma	46.9
Rio Verde	49.6
Russia-Barents Sea Coast	92.6
Russia-South East Coast	73.2
Sabarmati	34.9

Hydrologic Basin	Percent Positive Scenarios
Salinas Grandes	86.1
Salween	66.2
Sao Francisco	94.5
Saskatchewan-Nelson	69.7
Scandinavia-North Coast	76.7
Scheldt	91.2
Scotland	58.9
Seine	81.8
Senegal	53.3
Sepik	11.2
Shebelli-Juba	57.1
Siberia-North Coast	92.4
Siberia-West Coast	62
Sinai Peninsula	49.8
Sittang	59.2
Solomon Islands	10.1
South Africa-South Coast	92.7
South Africa-West Coast	88.5
South America-Colorado	82.8
South Argentina-South Atlantic Coast	79.8
South Atlantic Gulf	93.7
South Chile-Pacific Coast	89.4
South China Sea Coast	73.1
South Pacific Islands	10
Southern Central America	36.9
Spain-Portugal-Atlantic Coast	65.4
Spain-South and East Coast	46.8
Sri Lanka	65.2
St Lawrence	86.3
Sulawesi	92.4
Sumatra	79.4
Sweden	82.5
Syr Darya	49.2
Tagus	64.6
Taiwan	94.7
Tapti	48.3
Tarim Interior	85.3
Tasmania	90.1
Tennessee River	95

Hydrologic Basin	Percent Positive Scenarios
Texas Gulf Coast	92.6
Tiber	66.8
Tigris-Euphrates	28
Tocantins	67.6
Upper Colorado River	94.3
Upper Mississippi	90.8
Ural	42.1
Uruguay-Brazil-South Atlantic Coast	82.2
Viet Nam-Coast	43.9
Volga	86.5
Volta	16.5
Wisla	91
Xun Jiang	69.3
Yangtze	79.8
Yasai	1.3
Yenisey	76.4
Yucatan Peninsula	69.1
Zambezi	65.3
Ziya He-Interior	89.6

SI Table 1: Percent positive scenarios in each basin across the scenario ensemble.
